# Epigenetic control of macrophage polarization: implications for targeting tumor-associated macrophages

**DOI:** 10.18632/oncotarget.24556

**Published:** 2018-02-21

**Authors:** Amber E. de Groot, Kenneth J. Pienta

**Affiliations:** ^1^ The James Buchanan Brady Urological Institute, Johns Hopkins School of Medicine, Baltimore, MD, USA; ^2^ Department of Pharmacology and Molecular Sciences, Johns Hopkins School of Medicine, Baltimore, MD, USA; ^3^ Department of Oncology, Johns Hopkins School of Medicine, Baltimore, MD, USA; ^4^ Department of Chemical and Biomolecular Engineering, Johns Hopkins University, Baltimore, MD, USA

**Keywords:** macrophage, macrophage polarization, tumor-associated macrophage, epigenetics, tumor microenvironment

## Abstract

The progression of cancer is a result of not only the growth of the malignant cells but also the behavior of other components of the tumor microenvironment (TME). Tumor-associated macrophages (TAMs) are key components of the TME that influence tumor growth and disease progression. TAMs can either inhibit or support tumor growth depending on their polarization to classically-activated macrophages (M1s) or alternatively-activated macrophages (M2s), respectively. Epigenetic regulation plays a significant role in determining this polarization and manipulating the epigenetic regulation in macrophages would provide a means for selectively targeting M2s thereby eliminating tumor-supporting TAMs while sparing tumor-inhibiting M1 TAMs. Many pharmacologic modulators of epigenetic enzymes are currently used clinically and could be repurposed for treating tumors with high TAM infiltrate. While much research involving epigenetic enzymes and their modulators has been performed in M1s, significantly less is known about the epigenetic regulation of M2s. This review highlights the field’s current knowledge of key epigenetic enzymes and their pharmacologic modulators known to influence macrophage polarization.

## INTRODUCTION

Despite significant advancements over the years, cancer remains the second leading cause of death in the US with an estimated 600,920 cancer-related deaths and 1,688,780 new cancer cases in the US in 2017 [1]. As our understanding of the disease improves, it has become clear that the progression of disease is dependent not only on the growth of the malignant cells but also on the behavior of all components of the tumor microenvironment (TME). The interactions between tumor cells and the TME are analogous to ecosystem interactions and crosstalk between tumor cells and non-malignant cells within the TME create supporting networks that are critical for determining disease severity [2–5]. Despite the effectiveness of current therapies which focus on targeting malignant cells, patients continue to recur. Research has suggested that the TME plays a critical role in recurrence [6, 7]. Thus, cancer research has expanded to include efforts to target TME components and supportive networks [8, 9].

The TME includes various immune cells referred to by pathologists as tumor inflammation. While inflammation describes the presence of immune cells, it does not provide information as the how these cells influence the tumor. Multiple types of inflammation exist including Th1 responses which fight bacterial infections and Th2 responses which promote wound healing. The inflammatory response within a tumor depends on the types and activation states of infiltrating immune cells. While the type of tumor inflammation can be critical for prognosis, pathology reports do not differentiate between the inflammatory cell activation states, thus creating a gap in knowledge between the clinic and tumor immunology research.

The macrophage is one such immune cell that has drastically different effects on the tumor depending on its activation or polarization state [10]. Classically-activated macrophages, or M1s, drive Th1 responses and are generally polarized by stimulants such as INFγ, LPS, and other Toll-like receptor ligands. Markers and secreted molecules associated with an M1 polarization include STAT1, STAT3, IRF4, NFκB, CIITA, CD80, CD86, major histocompatibility complex class II receptor (MHC-II), chemokines receptors (CXCs), chemokine ligands (CXCLs and CCLs) including CCL2 (MCP1), COX-2, TNF, IFNγ, nitric oxide (NO), iNOS, reactive oxygen species (ROS), IL1β, IL12B, and IL6. Alternatively-activated macrophages, or M2s, drive Th2 responses and are generally polarized by IL4, IL13, IL10, and glucocorticoids. Markers and secreted molecules associated with an M2 polarization include SOCS1, PPARγ, STAT6, GATA3, c-MYC, HIF-1α, LXR, CD206 (MRC1), CD163, CD36, ARG1, IL10, TGFβ, matrix metalloproteases (MMPs), FIZZ1, and YM1 (Chi3l3). Macrophage phenotypes are plastic and it is recognized that macrophages exist across the polarization spectrum as they respond to new stimuli. Thus, the M1–M2 model of macrophage polarization is an acknowledged oversimplification as altering the expression of M1 markers does not necessitate the alteration of M2 marker expression and vice versa. Tumor-associated macrophages (TAMs) can exhibit either polarization phenotype and studies performed in multiple cancer types reveal a correlation between TAM polarization and prognosis [11–14]. Across all cancer types, higher M1 infiltrate correlates with a better prognosis and higher M2 infiltrate correlates with a poor prognosis.

Epigenetic regulation plays a significant role in controlling macrophage polarization and can be manipulated by pharmacologic modulators or inhibitors, many of which are used clinically against cancer and other diseases. Extensive work has been done to understand epigenetic regulation of macrophages in the context of infectious and chronic inflammatory diseases largely involving M1s. Several exceptional reviews have been published on these topics [15–18]. However, a current perspective of the epigenetic regulation relating to TAMs or M2s, especially in relation to cancer, remains an unmet need. This review aims to communicate the current knowledge of macrophage epigenetics applicable to TAMs and highlight the implications of these regulatory mechanisms for combating cancer.

### Epigenetic processes

Epigenetic regulation is the mechanism that allows genetically identical cells to differ phenotypically. This process is crucial for maintaining cell-type and tissue-specific functions and recapitulating them to daughter cells after replication. Conventional epigenetic mechanisms include DNA methylation, histone methylation, and histone acetylation which remodel chromatin to allow differential gene expression.

DNA methylation involves the methylation of the 5′-carbon on cytosine bases located in promoter CpG islands. This mark prevents transcriptional machinery from assembling on the altered promoter and silences gene transcription. Methyl groups are added to CpG islands by DNA methyl transferases (DNMTs) and removed by ten eleven translocation (TET) proteins.

Histone modification is important for activation state of both promoters and enhancers. Histones are modified on lysine and arginine residues on histone tails of primarily the H3 and H4 subunits. While histones can be modified with a number of post-translational modifications (PTMs) including phosphorylation and ubiquitination, the most important PTMs for controlling gene expression are methylation and acetylation. Histone acetylation marks promote gene transcription and are added to histones by histone acetyl transferases (HATs) and removed by histone deacetylases (HDACs). Histone acetylation marks are bound by bromodomain and extra-terminal motif (BET) proteins which initiate cellular processes such as transcription. Conversely, histone methylation marks can either activate or silence gene transcription depending on the residue modified, the number of methyl groups added to the residue, and the co-localization of the modified histone to an enhancer or promoter region of the regulated gene. Histone methylation is facilitated by histone methyl transferases (HMTs) and removed by histone demethylase (HDMs).

As histones remodel to expose enhancers, lineage-determining transcription factors (LDTFs) bind the open enhancers. These LDTFs determine a cell’s lineage and its response to various signals. When stimuli are applied, enhancer regions interact with promoter regions through bridges of scaffold proteins which include LDTFs and signal-dependent transcription factors (SDTFs) to initiate transcription. Well known LDTFs for myeloid cell lineages include PU.1 and C/EBP [15].

### Epigenetic regulation of macrophage polarization

**Figure 1:**
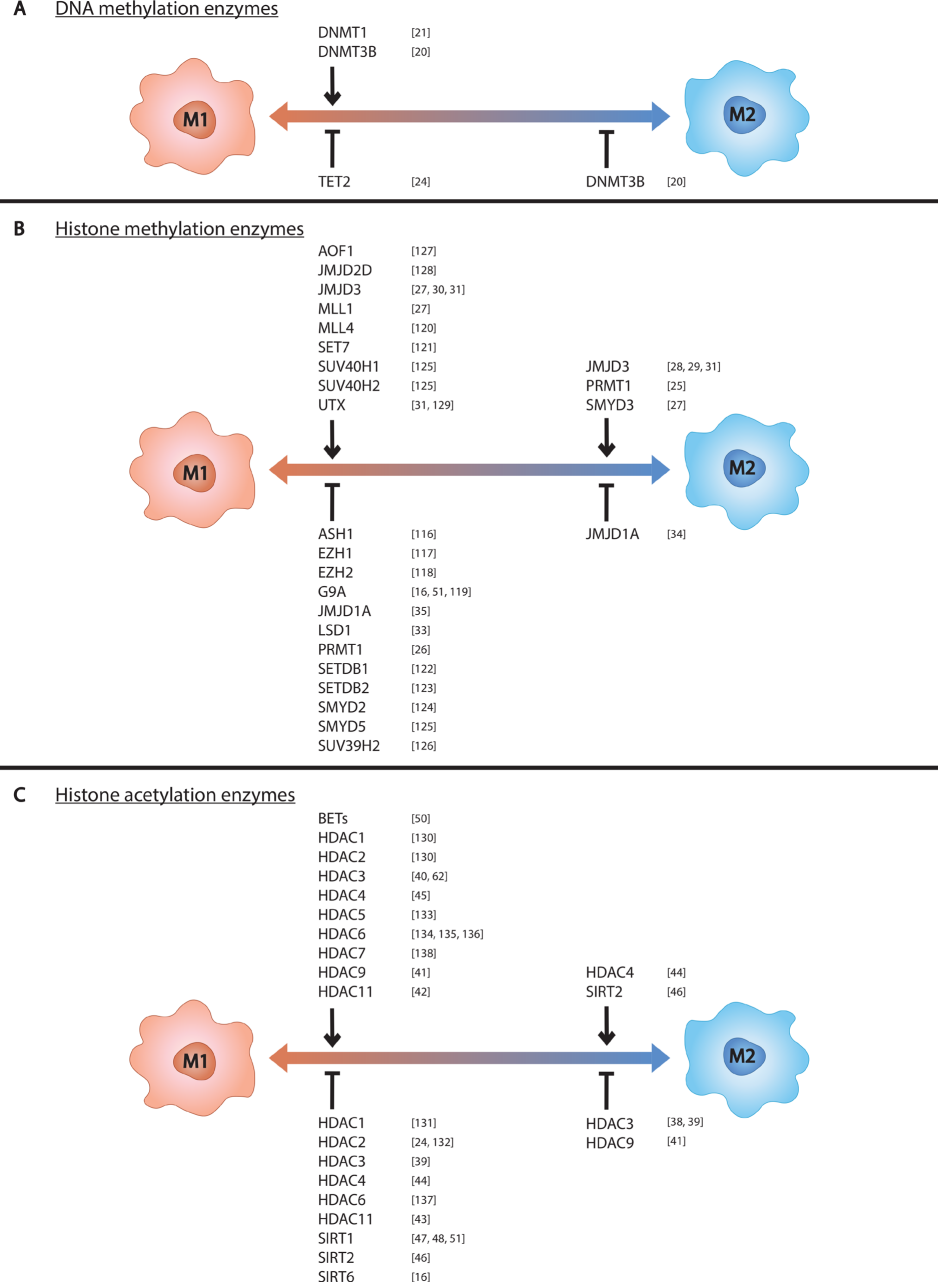
Epigenetic enzymes control macrophage phenotype Epigenetic enzymes known to control (**A**) DNA methylation, (**B**) histone methylation, and (**C**) histone acetylation in macrophages are listed above the left side of the spectrum if they promote M1 polarization and below if they inhibit M1 polarization. Those known to control M2 polarization are listed likewise on the right side of the spectrum.

While there exists a significant body of work on macrophage epigenetic processes, most of this work has been performed using M1s. Detailed below is the field’s current knowledge of epigenetic regulation in macrophages, emphasizing M2 and TAM biology. The impacts of these epigenetic regulators on macrophage phenotype are also listed in Table 1 and depicted in Figure 1.

**Table 1:** Effects of epigenetic enzymes on macrophage polarization to M1 or M2

	Epigenetic enzyme	Effect in M1s	Effect in M2s
DNMT	DNMT1	↓ SOCS1 [21]	
	DNMT3B	↑ TNF [20]	↑ Pparɣ promoter methylation [20] ↓ Arg1, CD206, Mgl-1, Pparg [20]
TET	TET2	↓ Il6 [24]	
HMT	ASH1	↓ Il6, Tnf [116]	
	EZH1	↓ IL6, TNF, IFNb, TLR signaling [117]	
	EZH2	↓ Ccl2, Ccl8 [118]	
	G9A (EHMT2)	↑ LPS tolerance [51] ↓ IL12B [119]; IFNb [16]	
	MLL1	↑ CXCL10 [27]	
	MLL4 (WBP7)	↑ Pigp, LPS signaling [120]	
	PRMT1	↓ CIITA [26]	↑ PPARɣ [25]
	SET7	↑ TNF, CCL2, IL8 [121]	
	SETDB1	↓ TNF [122]	
	SETDB2	↓ Cxcl1, Il12b, Cxcl2, Ym1 [123]	
	SMYD2	↓ Tnf, Il6, MHC-II, CD40/80 [124]	
	SMYD5	↓ Tnf, Il1a, Il1b, Ccl4, Cxcl10 [125]	
	SUV39H2	↓ IL6, TNF [126]	
	SUV40H1	↑ Tnf, Cxcl10 [125]	
	SUV40H2	↑ Tnf, Cxcl10, Phf2 [125]	
HDM	AOF1	↑ NFκB signaling [127]	
	JMJD2D	↑ Ccl22, Il12b [128]	
	JMJD3	↑ TNF [30]	↑ Arg1, Ym1 [28, 29]; Irf4, Fizz1 [28]; CD206 [29]
	LSD1	↓ Il6 [33]	
	UTX (KDM6A)	↑ IL6, IFNβ [129]	
HDAC	HDAC1	↑ IFN signaling, IRF3 activation [130] ↓ IL6 [131]	
	HDAC2	↑ IFN signaling, IRF3 activation [130] ↓ IL6 [24], MHC-II [132]	
	HDAC3	↑ IFNβ, Nos2, IL6 [40]; IL6, NO [62] ↓ TGFβ [39]	↓ IL4 signaling [38]
	HDAC4	↑ TNF, IL6 [45] ↓ NFκB signaling [44]	↑ STAT6 signaling, Arg1 [44]
	HDAC5	↑ TNF, CCL2, IL10 [133]	
	HDAC6	↑ LPS activation [134]; IL10 [135, 136] ↓ ROS [137]	
	HDAC7	↑ TLR signaling [138]	
	HDAC11	↑ antigen presentation, CD4+ T cell stimulation [42] ↓ IL10 [42]; IL1 [43]	
	SIRT1	↓ NFκB signaling [51]; Ccl2, Il1β, Il6, Nos2 [47]; TNF [47, 48]	
	SIRT2	↓ NFκB signaling, TNF, IL6, CCL2, IL1β [46]	↑ Gata3, Arg1, Cd11c [46]
	SIRT6	↓ IL1β [16]	
BET		↑ IL6, IL1b, IFNg, IL12B, Il1a, Ccl5, Cxcl10, Cxcl2/3 [50]	

### DNA methylation

As monocytes differentiate into macrophages, there is a net demethylation of promoter CpG islands [19]. Removing this silencing mechanism presumably primes these genes for transcription.

DNMT3B is the only DNMT with a known role in M2 differentiation and phenotypic control. Knockdown of DNMT3B in RAW264.7 macrophages and mouse bone marrow-derived macrophages (BMDMs) induces M2 polarization and prevents M1 marker expression, LPS-induced TNF secretion, and migratory response to CCL2 gradient [20]. Additionally, overexpression of DNMT3B prevents IL4-induced expression of *Arg1* [20]. DNMT3B also methylates the promoter of *Ppar*γ which is reduced in response to IL4 stimulation [20].

In M1s, DNMT1 positively regulates the M1 phenotype by silencing SOCS1 in LPS-stimulated RAW264.7 cells which induces TNF and Il6 expression [21]. If DNMT1 has similar suppressive functions of SOCS1 in M2s, then it likely suppresses the expression of M2 genes. However, functional studies of DNMT1 in M2s are required to implicate this enzyme as a negative regulator of M2 phenotype. Furthermore, DNMT1 is known to be the primary methyltransferase responsible for propagating DNA methylation marks during DNA replication [22]. This function suggests that DNMT1 has an essential role in regulating gene expression beyond SOCS1 in both M1s and M2s.

As for demethylation, TET2 loss of function mutations are implicated in myeloid malignancies, though little is known about the impact of TET proteins on macrophage polarization. Studies involving TET2 knockdown in mouse bone marrow-derived hematopoietic precursor cells revealed an increase in differentiation towards macrophage lineages implicating TET2 as a regulator of myelopoiesis [23]. Additionally, M1 polarization by LPS-stimulation of human and mouse macrophages upregulates expression of TET2 which acts as a co-repressor of *Il6* transcription alongside HDAC2 [24]. While this information is important for understanding myeloid- and M1-specific epigenetic regulation, it does not offer insight as to the role of TET2 or TET proteins in M2 polarization.

### Histone methylation

PRMT1 methylates the arginine located at residue 3 on the tail of histone H4 (H4R3) and is implicated as a positive regulator of M2 phenotype through its induction of PPARg in IL4-stimulated mouse peritoneal macrophages [25]. Alternatively, a study using IFNg-stimulated RAW264.7 cells revealed PRMT1 negatively regulates the M1 phenotype by repressing CIITA. Additionally, it was observed that PRMT1 is downregulated by IFNγ signaling [26]. Thus, PRMT1 adopts opposing roles in epigenetic regulation of M1s and M2s with its expression driving an M2 polarization.

Another HMT, SMYD3, a H3K4 methyltransferase, is speculated to positively regulate M2 polarization. Its expression levels increase in human monocyte-derived macrophages (HMDMs) with exposure to the combination of M-CSF, IL4, and IL13 (M-CSF + IL4 + IL13) and decrease with exposure to LPS + IFNγ [27]. Its upregulation coincides with methylation and transcriptional activation of ALOX15, a lipoxygenase M2 marker, which SMYD3 is known to regulate in other contexts [27]. However, functional studies are needed in order to make conclusions on these speculations about M2 phenotypic regulation. Additionally, no functional studies have been performed to test its role in M1 polarization.

JMJD3 (KDM6B), an H3K27 demethylase, has been recognized as an essential regulator of M2 polarization through its induction of *Irf4*, *Arg1*, *CD206*, and other M2 markers in IL4- stimulated [28] and IL4 + IL13-stimulated [29] mouse BMDMs. While this study revealed JMJD3 to be unnecessary for M1 polarization of TLR ligand-stimulated mouse peritoneal macrophages, other studies involving IFNg-stimulated HMDMs [27] or LPS + IFNγ-stimulated HMDMs from rheumatoid arthritis patients [30] detail its role in inducing pro-inflammatory cytokine expression. In addition to its upregulation in M2s, JMJD3 is also upregulated in IFNγ-stimulated HMDMs [27, 31]. Due to its positive regulatory role in both M1s and M2s, JMJD3 is attributed as a regulator of general stimulus response in macrophages rather than specific polarization roles [31]. Notably, loss of JMJD3 does not lead to significant changes in H3K27 methylation indicating that JMJD3 may regulate macrophage phenotype through modification of proteins other than histones [27, 31].

Though JMJD3 is the only HDM with a known effect on M2 polarization, LSD1 (KDM1A) and JMJD1A (KDM3A) play critical roles in myeloid cells. LSD1, a H3K4 and H3K9 demethylase, is essential for myeloid cell differentiation through silencing of stem and progenitor cell genes [32] and is also involved in LPS tolerance-induced Il6 silencing in mouse BMDMs [33]. Additionally, JMJD1A, a H3K9 demethylase, represses *Ccl2*, *Ccr1*, and *Ccr5* in mouse peritoneal macrophages and RAW264.7 cells under hypoxic conditions [34]. Furthermore, JMJD1A inhibition decreases macrophage infiltrate in subcutaneous A673 sarcoma tumors in mice [35]. These studies raise the possibility that JMJD1A plays a role in TAM biology and macrophage phenotype control especially in the context of a hypoxic tumor microenvironment.

### Histone acetylation

Certain acetylation marks have been discovered to contribute to macrophage phenotypic control. H3 acetylation is important for inducing *IFN*α, *TNF*, and *IL6* expression in THP-1 cells indicating the importance for H3 acetylation in expression of M1 phenotype [36]. More specifically, the H3K9 and H3K14 acetylation of *Tnf*, *Il6*, *Nos2*, and MHC-II promoters in LPS-stimulated mouse microglia is integral to expression of these genes [37]. However, the importance of these marks on M2 phenotype in IL4- or IL13-stimulated macrophages has yet to be elucidated.

While currently investigations into the importance of HATs in macrophage polarization are lacking, extensive observations have been made concerning the role of HDACs with some of these studies examining their effects in M2s.

HDAC3 negatively regulates M2 polarization by repressing IL4 signaling in mouse BMDMs [38] and TGFβ production in mouse peritoneal macrophages [39]. HDAC3 performs an opposing function in M1s by promoting M1 polarization in LPS-stimulated mouse BMDMs thereby proving critical for LPS signaling [40].

HDAC9 is another negative regulator of M2 polarization as peritoneal macrophages from HDAC9-deficient mice expressed higher levels of M2 genes and lower levels of M1 genes compared to wild type mice [41]. Additionally, the promoter acetylation levels and expression levels of *Ppar*γ were significantly increased in the HDAC9-deficient mice. The observations implicate HDAC9 as a repressor of the M2 phenotype and inducer of the M1 phenotype with *Ppar*γ promoter deacetylation acting as a key component of its regulatory mechanism.

While HDAC11 has not been studied in M2s, its effects in M1s suggest it may act as a negative regulator of M2 phenotype. In RAW264.7 cells, HDAC11 represses IL10 expression [42]. Thus, if HDAC11 acts similarly in M2s as it does in M1s, then it likely opposes M2 polarization. Apart from its effect on IL10, HDAC11 also represses IL1β in LPS-stimulated mouse BMDMs [43] and promotes antigen presentation and CD4^+^ T cell stimulation in RAW264.7 cells [42] thereby remaining ambiguous as to whether it promotes or inhibits M1 polarization.

HDAC4 induces STAT6 signaling and *Arg1* expression in IL4-stimulated mouse bone-marrow derived dendritic cells implicating it as a positive regulator of M2 polarization [44]. In M1s, HDAC4 functions as both a negative and positive regulator. One study using LPS + IFNg-stimulated of mouse BMDMs revealed that HDAC4 inhibited NFκB signaling [44] while another study using the LPS-stimulated mouse microglial BV2 cell line revealed that HDAC4 induces TNF and IL6 secretion [45].

The HDAC SIRT2 acts as another positive M2 phenotype regulator by inducing *Gata3*, *Arg1*, and *Cd11c* expression in IL4-stimulated mouse BMDMs [46]. The same study discovered SIRT2 also represses the M1 phenotype by downregulating NFkB signaling and IL1b and TNF secretion in LPS-stimulated mouse BMDMs [46].

SIRT1 has been tested against M2s but was found to have no impact on M2 polarization in IL4-stimulated mouse BMDMs [47]. It does, however, decrease expression of various M1 markers in LPS + IFNγ-stimulated mouse BMDMs [47] and LPS-stimulated RAW264.7 cells [48].

General BET protein activity promotes inflammatory cytokine production in LPS-stimulated mouse BMDMs [49, 50], although further studies investigating BET proteins in macrophage polarization are lacking.

### Epigenetic enzymes as targets for disrupting M2 polarization

Of the enzymes described here, the most relevant for targeting TAMs are those that promote the M2 phenotype, namely PRMT1, JMJD3, HDAC4, SIRT2, and potentially SMYD3. Inhibiting these enzymes in TAMs would prevent these macrophages from polarizing to M2s and supporting the tumor. It is important to note that histone modifying enzymes have secondary functions and act on proteins other than histones as well. Much of their macrophage-polarizing function is speculated to arise from modification of non-histone proteins especially since many histone modifying enzymes are known to act in the cytoplasm [15, 51]. Additionally, not all regulators have opposing function in M1s vs M2s and not all regulators have exclusively positive or negative effects on one polarization state. These discrepancies highlight the complexity of macrophage biology and the challenges of finding effective epigenetic targets in TAMs. There are several enzymes that have known functional roles in M1s but are not discussed here because they have not been studied in M2s. These enzymes and their effects on M1 phenotype are included alongside the enzymes discussed above in Table 1 and Figure 1. This extensive list exemplifies the discrepancy between macrophage research in M1- and M2-related diseases. In order to progress our understanding of the TME’s influences on tumor growth, further investigations into mechanisms of M2 phenotypic control are needed.

### Pharmacologic modulators and their effects in macrophages

**Figure 2:**
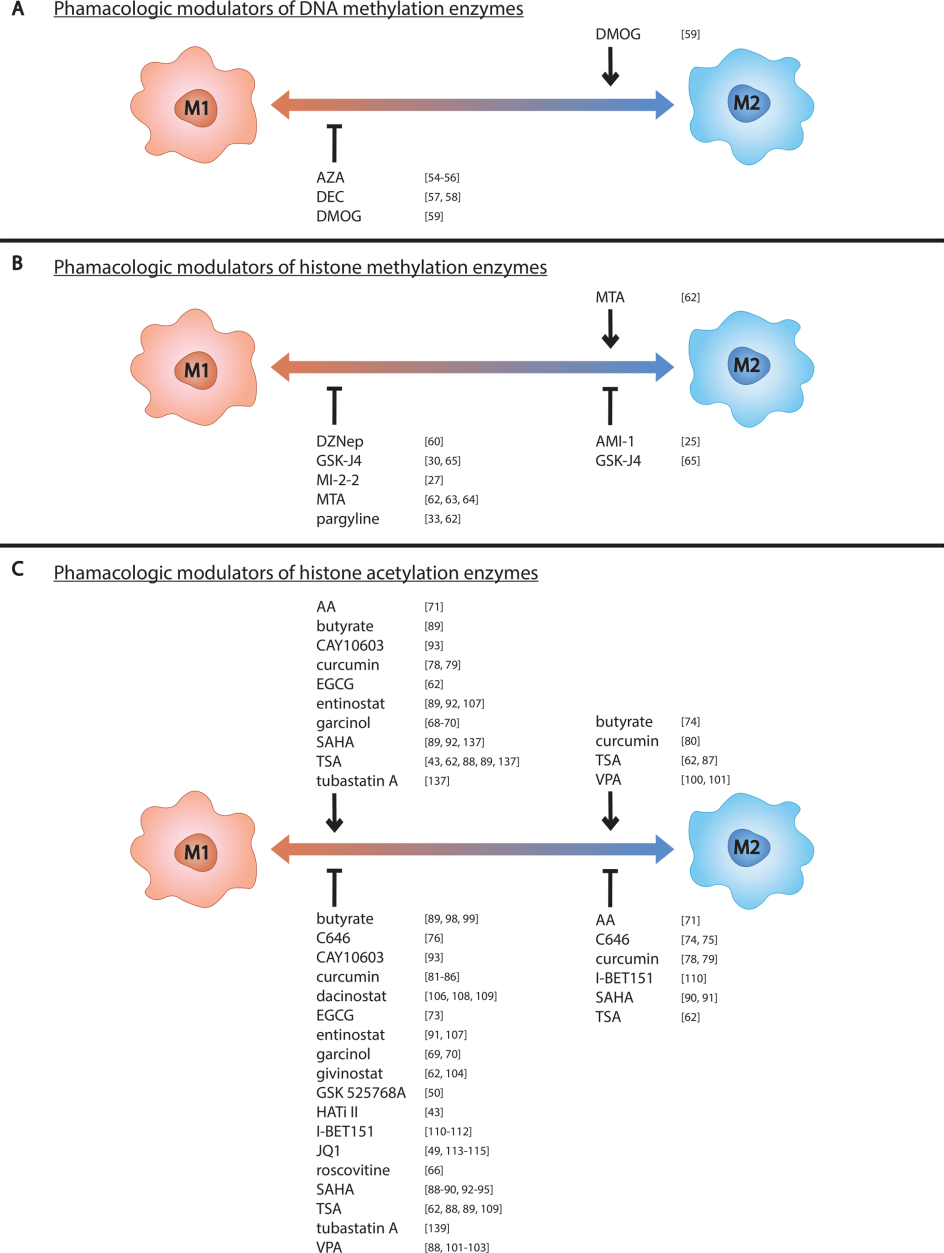
Pharmacologic modulators of epigenetic enzymes influence macrophage phenotype Pharmacologic modulators known to influence (**A**) DNA methylation, (**B**) histone methylation, and (**C**) histone acetylation in macrophages are listed above the left side of the spectrum if they promote M1 polarization and below if they inhibit M1 polarization. Those known to control M2 polarization are listed likewise on the right side of the spectrum.

There are many pharmacologic modulators of epigenetic enzymes, some of which target specific enzymes and others which broadly target multiple enzyme classes. Modulators of inhibitors that target DNMTs are commonly referred to as DNMTis, those that target TET proteins are referred to as TETis, and so on. Not all epigenetic modulators have been tested against macrophages and even less have been tested against M2s specifically. Discussed below are the pharmacologic modulators tested against macrophages and their effect on macrophage polarization. This information is also listed in Tables 2 and 3 and depicted in Figure 2.

**Table 2:** Effects of pharmacologic modulators of DNA methylation on macrophage polarization to M1 or M2

	Pharmacologic modulator	Effect in M1s	Effect in M2s
DNMTi	azacytidine (Vidaza^®^, AZA)	↑ Arg1, Fizz1 [55] ↓ iNOS, NO [54–56]; TNF [56]	
	decitabine (Dacogen^®^, DEC)	↑ ARG1, CD206, STAT3 activation [58]; SOCS1 [21]; PPARɣ, LXRα [57] ↓ TNF, IL6, IL1β, iNOS, CCL2, CCL5 [57, 58]; CCL9 [57]; IL1α, CCL3, CCL4, CCL7, CCL10, CCL12, IL1RN [58]	
TETi	dimethyloxallyl glycine (DMOG)	↑ IL10 [59] ↓ NFκB activity, iNOS [59]	↑ Arg1, Fizz1, Ym1 [59]

**Table 3:** Effects of pharmacologic modulators of histone modification on macrophage polarization to M1 or M2

	Pharmacologic modulator	Effect in M1s	Effect in M2s
HMTi	3-deazaneplanocin (DZNep)	↓ TNF [60]	
	AMI-1		↓ PPARɣ, CD36, CD206, CD209, SOCS1 [25]
	methylthioadenosine (MTA)	↑ Il1β [62] ↓ TNF [62, 64]; IL6 [62]; iNOS [64]; CD69, CD86, MHC-II, NFκB signaling [63]	↑ Arg1 [62]
	MI-2-2	↓ CXCL10 [27]	
HDMi	GSK-J4	↓ TNF [30, 65]	↓ CD206 [65]
	pargyline (Eutonyl, Eutron)	↓ TNF [62]; LPS tolerance-induced Il6 silencing [33]	
HATi	C646	↓ NFκB activation, TNF, IL8, IL12, iNOS, IL1β [76]	↑ Ym1, Cd36 [75] ↓ Fizz1, Mgl2 [75]; Arg1 [74, 75]; Ym1 [74]
	curcumin	↑ PPARɣ, CD36 [82]; SOCS-1, SOCS-3 [84] ↓ TNF, IL6 [81, 82, 84]; IL12B [81, 82]; CCL2 [83, 85]; NFκB activation [81]; ROS [85], COX-2 [84]	↑ IL12 [78] ↓ IL10, TGF-β, MMP2, MMP9, VEGF, STAT3 activation [78]
	epigallocatechin-3-gallate (EGCG)	↑ Tnf [62] ↓ TNF [73]	
	garcinol	↑ TNF, IL6 [68–70] ↓ COX-2, iNOS [69, 70]; NFκB, NO [69]	
	Histone Acetyltransferase Inhibitor II (HATi II)	↓ IL1β [43]	
	roscovitine	↓ COX-2, iNOS, NO, NFκB activation [66]	
HDACi	butyrate	↑ Cox-2 [89] ↓ Ccl7 [89]; NO, IL6, IL12B [98]	
	CAY10603 (BML-281)	↑ TNF, IL1β [93] ↓ IL12B, IL6 [93]	
	dacinostat (LAQ824)	↓ TNF, NFκB activation [109]; MCP-2, MCP-3, CCL2, CCL15, CCL23, CCR1, CCR5, CD38 [108]; IL10 [106]	
	entinostat (MS-275)	↑ TNF, IL1β, IL12B, NFκB activity [92]; Cox-2 [89]; IL10 [92, 107] ↓ IL1β, iNOS, IFNɣ, IL17, MMP-9 [107]	
	givinostat (ITF2357)	↓ NO [62, 104]; IL6 [62]; TNF [104]	
	trichostatin A (TSA)	↑ Cox-2 [89]; Stat1 [62]; IL1β [43]; IFNβ [88]; ROS [137] ↓ Tnf [62, 88, 109]; Il6 [62, 88]; Cox-2 [62]; NFκB activation [109]; Ccl7 [89]; IL12B [88, 89]; Ccl8, Ccl12, Cxcl10, Nos2, Irf7 [88]	↑ c-Myc [87]; Cdh1 [62] ↓ Arg1, Fizz1, Ym1 [62]
	tubastatin A	↑ ROS [137] ↓ phagocytosis [137]; TNF, IL6, NO [139]; apoptosis [105]	
	valproic acid (Depakene®, VPA)	↑ IL10, CD86 [102] ↓ CD40, CD80 [102]; IL12B [88, 102]; iNOS, TNF, IL6 [88]	
	vorinostat (Zolinza®, SAHA)	↑ Cox-2 [89]; Il1β [92]; ROS [137] ↓ NO [90]; TNF, IL6, IL12B [88]; Ccl7 [89]	↓ TAM infiltration in tumors [90, 91]
BETi	GSK 525768A (I-BET-762)	↓ Il6, Ifnβ, Il12α, Cxc19, Ccl12 [50]	
	I-BET151	↑ PPARɣ, LXR [112] ↓ IL6 [111]; NFκB signaling [112]; CXCL10, CXCL11, IFNβ [110]	↓ PPARɣ, ENPP2, MS4A4A, IL7R, ABIN3 [110]
	JQ1	↓ TNF, IL6 [49, 113]; CCL2 [49]; Il1β [113]; Nos2 [114]; PD-L1 [115]	

### DNMTis

Azacytidine (AZA) and decitabine (DEC), otherwise known as Vidaza^®^ and Dacogen^®^, respectively, are two well-established DNMTis used clinically to treat myelodysplastic syndrome (MDS) [52, 53]. Both are involved in clinical trials against various leukemias and solid tumors (Table 4) [52, 53]. The effects of AZA and DEC have not been investigated in M2s although many studies have examined the effects of these modulators in M1s and unpolarized macrophages.

**Table 4:** Clinical applications of pharmacologic modulators tested against macrophages

	Pharmacologic modulator	Approved clinical indications ^a^	Clinical trial cancer indications (total # of trials for cancer) ^b^
DNMTi	azacytidine (Vidaza^®^, AZA)	acute myeloid leukemia, chronic myelomonocytic leukemia	various hematologic and solid tumor cancers (520)
	decitabine (Dacogen^®^, DEC)	acute myeloid leukemia, chronic myelomonocytic leukemia, myelodysplastic syndrome	various hematologic and solid tumor cancers (241)
TETi	dimethyloxallyl glycine (DMOG)		
HMTi	3-deazaneplanocin (DZNep)		
	AMI-1		
	methylthioadenosine (MTA)		lung cancer and sarcoma (2)
	MI-2-2		
HDMi	GSK-J4		
	pargyline (Eutonyl, Eutron)	hypertension c	
HATi	anacardic acid (AA)		
	C646		
	curcumin		various hematologic and solid tumor cancers (55)
	epigallocatechin-3-gallate (EGCG)		various solid tumor cancers (28)
	garcinol		
	Histone Acetyltransferase Inhibitor II (HATi II)		
	roscovitine		breast cancer, non-small cell lung cancer, advanced solid tumors (3)
HDACi	butyrate		various hematologic and solid tumor cancers (114)
	CAY10603 (BML-281)		
	dacinostat (LAQ824)		
	entinostat (MS-275)		various hematologic and solid tumor cancers (47)
	givinostat (ITF2357)		various hematologic cancers (6)
	trichostatin A (TSA)		various hematologic and solid tumor cancers (8)
	tubastatin A		
	valproic acid (Depakene^®^, VPA)	epilepsy, migraine, schizophrenia, seizures, acute manic episodes	various hematologic and solid tumor cancers (82)
	vorinostat (Zolinza^®^, SAHA)	cutaneous T-cell lymphoma	various hematologic and solid tumor cancers (242)
BETi	GSK 525768A (I-BET-762)		breast cancer, prostate cancer, hematologic cancers, NUT midline carcinoma (4)
	I-BET151		
	JQ1		

^a^Information acquired from www.drugbank.ca.

^b^Information acquired from clinicaltrials.gov.

^c^All FDA-approved drugs have been discontinued [140].

In M1s, AZA decreases M1 marker expression and increases M2 marker expression. AZA treatment lowers iNOS expression and NO production in LPS- or PGN-stimulated RAW264.7 cells [54], PGN-stimulated mouse BMDMs [55], and mouse peritoneal macrophages stimulated with LPS and IFNγ [56]. The mouse peritoneal macrophages also exhibited decreased TNF expression [56]. Additionally, in the PGN-stimulated RAW264.7 cells, AZA increased expression of M2 markers *Arg1* and *Fizz1* [55]. A similar effect was observed in mouse macrophages from a myocardial infarction mouse model with cardiac macrophages from untreated mice highly expressing iNOS while cardiac macrophages from AZA-treated mice highly expressing ARG1 [55].

DEC has similar effects to AZA in M1s and unstimulated macrophages. DEC lowers expression of M1 markers TNF, IL6, IL1a, IL1b, iNOS, and a number of chemokines including CCL2, CCL5, and CCL9 in various LPS-stimulated macrophage models and macrophages from atherosclerotic plaques in *Ldl*-deficient mice [57, 58]. DEC also increases expression of the M2 markers ARG1 in lung macrophages from LPS-treated mice and CD206 in LPS-stimulated mouse BMDMs [58]. This study also found the effects of DEC on *Nos2*, *Arg1*, and *CD206* expression to be accentuated when in combination with trichostatin A (TSA), an HDAC inhibitor [58]. Additionally, when used in combination with TSA in LPS-stimulated mouse BMDMs, DEC increases STAT3 phosphorylation which downregulates the M1 inflammatory response [58]. Furthermore, DEC increases promoter demethylation and expression of SOCS1 in LPS-stim RAW264.7 cells which promotes the STAT3 pathway [21] and PPARγ and LXRα in unstimulated RAW264.7 cells which causes a decrease in M1 markers TNF, IL6, CCL2, and CCL5, an effect that is reversed by PPARγ and LXRα knockdown [57].

Taken together, these studies suggest that these DNMT modulators would likely promote the M2-TAM phenotype if used in the context of cancer therapy, though no definitive conclusions can be made due to the lack of studies of these modulators in M2s or TAMs.

### TETis

The TET protein inhibitor dimethyloxallyl glycine (DMOG) promotes M2 polarization by upregulating *Arg1*, *Fizz1*, and *Ym1* in mouse peritoneal macrophages *in vivo* when exposed to chitin, an M2 stimulatory molecule, or *in vitro* when exposed to IL10 or IL4 + IL13 [59]. In M1s, DMOG downregulates the M1 phenotype by inhibiting LPS-induced NFkβ activity and iNOS expression but upregulates M2 marker IL10 expression [59]. Notably, in unstimulated mouse peritoneal macrophages, DMOG increases expression of the M1 marker iNOS as well as NFkβ activity [59]. These findings are significant for understanding the role of TET proteins in macrophages but suggest that using DMOG in a tumor would promote M2 polarization of TAMs.

### HMTis

Few HMTis have been tested against macrophages. 3-Deazaneplanocin (DZNep), an EZH2 inhibitor, inhibits TNF expression in LPS-stimulated RAW264.7 cells [60]. MI-2-2, an inhibitor of MLL-Menin interactions, decreases CXCL10 expression in IFNg-stimulated HMDM [27]. While these inhibitors downregulate M1 phenotype, they have not been tested against M2s.

On the other hand, methylthioadenosine (MTA), an HMT inhibitor, currently involved in clinical trials for oral health [61], has been tested against M2s. In IL4-stimulated mouse BMDMs, pretreatment with MTA upregulated the M2 phenotype by increasing *Arg1* expression although it did not significantly affect M2 markers *Fizz1*, *Ym1*, *Cdh1*, and *CD206* [62]. The same study found pretreatment with MTA partially downregulated the M1 phenotype by decreasing *Tnf* mRNA levels as well as TNF and IL6 secretion in LPS + INFγ-stimulated mouse BMDMs while increasing *Il1b* mRNA levels but exerting no effect on *Il6*, *Nos2*, and *Stat1* mRNA levels [62]. Other studies which test MTA against M1s have found the same compound downregulates the M1 phenotype in IFNg-stimulated RAW264.7 cells [63] and LPS-stimulated mouse BMDMs [63, 64]. Though MTA has not been tested with *in vivo* tumor models, its promotion of the M2 phenotype makes it an unlikely candidate for targeting M2 TAMs.

AMI-1, a PRMT1 inhibitor [25], downregulates the M2 phenotype in M2s by decreasing expression of PPARγ and PPARγ-dependent genes CD36, CD206, CD209, and SOCS1 during IL4-induced differentiation of mouse peritoneal macrophages [25]. Though no studies have been performed with M1s, this is a promising compound for altering TAM phenotype since it inhibits normal M2 processes and thus carries the potential to attenuate immunosuppressive, tumor-promoting function of M2-TAMs.

### HDMis

Two HDMis, pargyline and GSK-J4, have been tested against macrophages, including M2s. Pargyline, a monamine oxidase and pan-HDM inhibitor previously sold as Eutonyl or Eutron, did not significantly affect the expression of M2 markers *Arg1*, *Fizz1*, *Ym1*, *Cdh1*, and *CD206* in IL4-stimulated mouse BMDMs. Alternatively, the same study found that although pargyline decreases TNF secretion in LPS-challenged mouse BMDMs, it does not significantly affect expression of M1 markers *Tnf*, *Il6*, *Nos2*, *Stat1*, and *Il1b* in IFNγ + LPS-stimulated mouse BMDMs [62]. Thus, it appears that pargyline has weak control over macrophage polarization. A separate study found that pargyline prevents LPS tolerance-induced *Il6* silencing in mouse BMDMs [33].

GSK-J4, which targets JMJD3 and other KDM6 enzymes, reduces CD206 expression in IL4-stimulated HMDMs [65] and inhibits expression of TNF and other M1 inflammatory cytokines in LPS-stimulated [30] and IFNγ-stimulated [65] HMDMs. These effects are consistent with expectations for a JMJD3 inhibitor due to JMJD3’s vital role in both M1 and M2 polarization. Regardless of its effects on M1s this inhibitor may be a promising compound for decreasing the immunosuppressive and tumor-promoting functions of TAMs.

### HATis

Several HATis have been tested in M1s or unstimulated macrophages with various effects observed to the M1 phenotype. The M1 phenotype was downregulated in M1s by Histone Acetyltransferase Inhibitor II (HATi II) which decreased IL1b secretion [43] and by roscovitine which decreased COX-2 and iNOS expression, NO production, and NFκB activation [66]. However, roscovitine, a p300 inhibitor, is better known for its CDK-inhibiting functions [67]. Thus, these effects on macrophage polarization may be due to its influence on cell cycle regulation rather than epigenetic regulation. Garcinol, another p300 inhibitor, has mixed results on M1s; it increases TNF and IL6 expression while decreasing iNOS expression, NO production, COX-2 expression, and NFκB activation in LPS-stimulated RAW264.7 cells [68–70]. Another HATi, anacardic acid (AA), has a negative effect on the M2 phenotype as it decreases IL4 and IL10 secretion when tested against unstimulated macrophages [71]. In these same cells, it has mixed effects on the M1 phenotype as it increases NFκB phosphorylation, migration, phagocytosis, and secretion of NO, IL6, and TNF [71]. Though these results are informative for a number of inflammatory diseases, none of these HATis have been tested against M2s. Therefore, inferences as to these modulators’ efficacy against TAMs cannot be drawn with the potential exception of AA which appears to inhibit the M2 phenotype.

Currently, only three HATis have been tested against M2s: epigallocatechin-3-gallate (EGCG), C646, and curcumin. EGCG, which is involved in a number of clinical trials for indications such as type 2 diabetes, obesity, and various cancers [72], does not significantly affect expression of M2 markers *Arg1*, *Fizz1*, *Ym1*, *Cdh1*, and *CD206* in IL4-stimulated mouse BMDMs [62]. Additionally, its effects on M1s are ambiguous. The same study found that EGCG increases *Tnf* expression without affecting expression of *Il6*, *Nos2*, *Stat1*, and *Il1b* in LPS + IFNγ-stimulated BMDMs [62] whereas another study found EGCG inhibits TNF expression in LPS-stimulated HMDMs [73]. Due to this ambiguity and lack of effect on M2 polarization, EGCG exhibits little promise as a targeting agent against M2 TAMs.

Studies implicating C646, a second p300 inhibitor, in macrophage polarization have produced slightly more conclusive results. In M-CSF + IL4-stimulated mouse BMDMs, C646 downregulates *Arg1* and *Ym1* expression [74]. Another study also found C646 downregulates *Arg1*, *Fizz1*, and *Mgl2* expression but conversely upregulates *Ym1* and *CD36* in IL4-stimulated mouse BMDMs [75]. In M1s, C646 decreases NFkB activation and expression of TNF, IL8, IL12B, iNOS, and IL1b [76]. C646’s effects on M2s is somewhat ambiguous but suggests that it might decrease the tumor-promoting functions of M2 TAMs within the context of a tumor.

Curcumin is another p300 inhibitor that is often used therapeutically for various cancers, parasitic infections, inflammatory diseases, & other indications [77]. In a study involving nanoparticles containing the synthetic curcumin derivative hydrazinocurcumin (HC), HC exposure altered the phenotypes of RAW264.7 cells previously co-cultured with 4T1 breast cancer cell line cells. After co-culture but before HC exposure, the RAW264.7 cells exhibited an IL10^hi^, IL12^lo^, TGF-β^hi^ M2-like phenotype with high STAT3 activity and high expression of STAT3 downstream genes MMP2, MMP9, and VEGF. After HC exposure, these cells exhibited an IL10^lo^, IL12^hi^, TGF-β^lo^ M1-like phenotype with low STAT3 activity and expression of STAT3 downstream genes MMP9, MMP2, and VEGF [78]. These HC-exposed RAW264.7 cells also decreased 4T1 cell proliferation and migration when co-cultured and prolonged survival and reduced tumor burden when administered *in vivo* to mice co-injected with RAW264.7 cell and 4T1 cells subcutaneously [78]. Others have observed results similar to this with increased levels of *Stat4* and *Il12* as well as decreased levels of *Stat3*, *Il10*, and *Arg1* in tumor and spleen tissue of mice with subcutaneous 4T1 breast cancer cell line tumors suggesting a shift from M2- to M1-prominent TAM populations [79]. This study also observed reduced tumor volume and weight with curcumin treatment. Notably, this study analyzed cytokine secretion of whole tissue rather than isolated macrophages, therefore it cannot be concluded that the change in cytokine levels is due to a shift in macrophage phenotype. However, studies using unstimulated macrophages also found curcumin promoted an M2 phenotype with curcumin-treated RAW264.7 cells expressing higher levels of IL4, IL13, CD206, ARG1, PPARg, and phosphorylated STAT6 [80]. Alternatively, studies testing curcumin against M1s show it decreases M1 phenotype by downregulating NFκB activity, ROS production, and expression of TNF, IL6, IL12B, CCL2, and COX-2 while increasing expression of the M2 markers PPARγ and CD36 [81–85]. Similar observations have been made in renal tissue of mice with daunorubicin-induced nephrotoxicity [86]. *In vivo* curcumin treatment in this model increased M2 markers Il10, CD163, and CD36 while decreasing M1 markers CD80, CD86, IFNγ, IL6, TNF, TNF-R1, COX-2, and ICAM-1 expression and NFκB activation [86]. While various macrophage studies and models produce deviating effects, the *in vivo* tumor model experiments suggest that curcumin may be an effective pharmacologic modulator for targeting M2 TAMs.

### HDACis

HDACis are the most extensively studied epigenetic modulator in macrophages, however, nearly all of this work has been performed in M1s in the context of infectious or inflammatory disease. Trichostatin A (TSA) is one of a few HDACis tested against M2s. In IL4-stimulated mouse BMDMs, TSA inhibited *Arg1*, *Fizz1*, and *Ym1* expression while increasing *Cdh1* expression without affecting *Cd206* expression [62]. Additionally, it was found to upregulate *c-Myc*, an important regulator of the M2 phenotype, in GM-CSF-stimulated mouse BMDMs [87]. Because of these opposing effects on M2 phenotype, it is difficult to predict whether TSA would successfully target M2s in the tumor. TSA has mixed effects in M1s across multiple studies reporting decreased expression of some M1 markers such as *Tnf* and *Il6* with increased expression of other markers such as *Stat1*, IFNb, and a number of chemokine ligands [62, 88]. Additionally, a number of these studies report conflicting results regarding *Cox-2*, IL1b, and iNOS expression [43, 62, 88, 89]. These discrepancies in TSA’s influence on LPS-induced effect may be attributed to variations in concentration as has been reported [89]. As mentioned previously, the immunomodulatory effects of TSA in M1s is enhanced by combination treatment with the DNMTi AZA [58].

Another prominent pan-HDACi, vorinostat, otherwise known as Zolinza^®^ or suberoylalanide hydroxamic acid (SAHA), is clinically used to treat cutaneous T cell lymphoma (Table 4). SAHA has yet to be tested using *in vitro* M2 models but has been used in mouse tumor models. In PyMT mice, SAHA delays tumor growth and reduces tumor burden and inhibits TAM infiltration of estrogen receptor-negative (ER^-^) mammary tumors while decreasing M-CSF and MMP-9 levels in these tumors [90]. Additionally, SAHA inhibits the increase of F4/80^+^ and ARG1^+^ macrophages in mouse pancreatic cancer tumors [91] making it a promising agent for targeting M2 TAMs. SAHA has been studied much more extensively in M1 models and has been found to generally inhibit LPS- and IFNγ-induced signaling and polarization [90, 92–95].

Butyrate, a pan-HDACi involved in clinical trials for schizophrenia [96], increases expression of M2 markers *Arg1*, *Fizz1*, *Ym1*, and CD206 & STAT6 phosphorylation in unstimulated mouse BMDMs [74]. Additionally, oral administration reduces progression of atherosclerosis by reducing migration and adhesion of macrophages [97]. Experiments using *in vitro* M1 models found decreased LPS-induced *Ccl7* and pro-inflammatory mediator expressions in butyrate-treated M1s [89, 98]. Butyrate also decreases expression of M1 markers NO, IL12B, and IL6 without affecting TNF or CCL2 in RAW264.7 cells co-cultured with 3T3-L1 adipocytes [99]. Because it generally inhibits the M1 phenotype and promotes the M2 phenotype, it would likely fail as a M2 TAM-targeting agent.

Valproic acid (VPA), also known as Depakene^®^, is another pan-HDACi used to clinically treat seizure disorders, mania, and migraine headaches and is involved in multiple clinical trials for neurological indications (Table 4). Its effects on M2s have not been elucidated although it has been shown to inhibit tumor growth enhancement induced by decoy receptor 3 which promotes M2 TAM infiltration [100]. Additionally, in a nitrogen mustard-induced lung injury model, VPA decreases iNOS^+^CCR2^+^ macrophages and increases CD68^+^, CD163^+^, and ATR-1a^+^ macrophages in lung tissue [101]. In M1 *in vitro* models, VPA has been shown to generally decrease M1 markers and phenotype [88, 102, 103]. However, VPA’s promotion of M2 polarization *in vivo* makes it a poor M2 TAM-targeting agent.

Several other selective HDACis and pan-HDACis including CAY10603 (BML-281), dacinostat (LAQ824), entinostat (MS-275), givinostat (ITF2357), and tubastatin A have been tested against macrophages, however, these have only been tested against M1s. Givinostat, tubastatin A, and CAY10603 decrease the pro-inflammatory phenotype [62, 93, 104, 105] while entinostat and dacinostat both reduce and enhance the M1 phenotype [89, 91, 92, 106–109]. Additionally, of note, dacinostat decreases IL10 expression in LPS-stimulated mouse peritoneal macrophages [106].

### BETis

Of the three BETis tested against macrophages, only I-BET151 has been tested against M2s. I-BET151 inhibited expression of M2 marker genes PPARγ, ENPP2, MS4A4A, IL-7R, and ABIN3 in HMDMs stimulated with IL4 or IL10 [110] implicating it as a promising M2 TAM-targeting agent. In M1s, I-BET151 decreases M1 marker expression [110–112] similar to the other BETis tested against M1s, GSK525768A (I-BET-762) [50] and JQ1 [49, 113–115].

### Epigenetic modulators as TAM-targeting agents

Of the pharmacologic modulators described, the most promising as M2 TAM-targeting agents are those that inhibit M2 polarization or decrease TAM infiltrate, namely, HMTi AMI-1; HDMi GSK-J4; HATis C646, curcumin, and potentially AA; HDACis SAHA and potentially TSA; and BETi I-BET151. Many epigenetic modulators have been and are being tested in clinical trials to treat various cancers. These modulators are listed in Table 3 alongside their approved clinical indications and clinical trial indications. While these pharmacologic modulators undoubtedly target the malignant cells, they also likely impact the rest of the tumor microenvironment including TAMs. The effect of these epigenetic modulators on the TME as a whole and TAMs in particular remains largely undetermined and it is possible that a significant portion of their anti-tumor effect is a result of modulating TAM tumor-promoting support networks. Understanding pharmacologic modulators in the context of their effect on all cells within the tumor microenvironment may expand their scope as anti-cancer agents. Their indications, for example, could be expanded to include cancers that are supported by TAM infiltration and not just cancers with significant epigenetic dysregulation.

## CONCLUSIONS

The epigenetic mechanisms controlling macrophage polarization are complex. There has been much progress in elucidating these mechanisms in inflammatory macrophages but TAMs have largely been overlooked. It is clear that pharmacologic modulators of epigenetic enzymes have effects that are not cell-specific affecting all cells in the TME and throughout the body and conferring therapeutic effects as well as different levels of toxicity. However, many of these modulators have already been tested clinically and deemed safe for therapeutic use. Therefore, epigenetic modulators provide a promising method for targeting TAMs and, due to their current clinical availability, can easily be repurposed for cancers with high M2 TAM infiltrate. Exploiting the differences in M1 and M2 biology using these modulators would provide a means for targeting M2 TAMs thereby eliminating these key tumor-supporting cells from the TME.

## Abbreviations

AZA: azacytidine
BET: bromodomain and extra-terminal motif
BETi: BET protein modulator/inhibitor
BMDM: bone marrow-derived macrophage
DEC: decitabine
DNMT: DNA methyltransferase
DNMTi: DNMT modulator/inhibitor
EGCG: epigallocatechin-3-gallate
HATi: HAT modulator/inhibitor
HATi II: Histone Acetyltransferase Inhibitor II
HC: hydrazinocurcumin
HDAC: histone deacetylase
HDACi: HDAC modulator/inhibitor
HDM: histone demethylase
HDMi: HDM modulator/inhibitor
HMDM: human monocyte-derived macrophage
HMT: histone methyltransferase
HMTi: HMT modulator/inhibitor
LDTF: lineage-determining transcription factor
MHC-II: major histocompatibility complex class II receptor
MMP: matrix metalloprotease
NO: nitric oxide
PTM: post-translational modification
ROS: reactive oxygen species
SAHA: suberoylalanide hydroxamic acid (otherwise known as vorinostat or Zolinza^®^)
SDTF: signal-dependent transcription factor
TAM: tumor-associated macrophage
TET: ten eleven translocation
TETi: TET protein modulator/inhibitor
TME: tumor microenvironment
TSA: trichostatin A
VPA: valproic acid
